# Basal cell carcinoma of the umbilicus associated with sweat gland structures: Two case reports

**DOI:** 10.1097/MD.0000000000033128

**Published:** 2023-02-22

**Authors:** Yukiho Kurosaki, Saori Itoi-Ochi, Asako Ota, Akiko Miyazaki, Hiroko Kato, Satoshi Nojima, Eiichi Morii, Kazunori Yokoi, Manabu Fujimoto, Atsushi Tanemura

**Affiliations:** a Department of Dermatology, Suita Municipal Hospital, Osaka, Japan; b Laboratory of Advanced Cosmetic Science, Osaka University Graduate School of Pharmaceutical Sciences, Osaka, Japan; c Department of Pathology, Osaka University Graduate School of Medicine, Osaka, Japan; d Department of Dermatology, Osaka University Graduate School of Medicine, Osaka, Japan.

**Keywords:** basal cell carcinoma, immunohistochemistry, sweat glands, umbilicus

## Abstract

**Rationale:**

Basal cell carcinoma (BCC) arising in the umbilicus is relatively rare, and in particular, there have been few reports mentioning peritumoral sweat gland structures histopathologically. We herein, report 2 cases of umbilical BCC with sweat gland structures within and around the tumor.

**Patient concerns:**

A 61-year-old woman had a 2-year history of black exudative plaque in her umbilicus, and an 80-year-old woman had a 6-month history of dark brownish plaque in the umbilicus, with exudation 2 months prior to her first visit.

**Diagnoses:**

Based on the histopathological finding, both cases were confirmed as BCC. The results of immunohistochemical staining showed that the tumor cells were Ber-EP4 positive. In addition, EMA-positive glandular structures were seen within and around the tumor.

**Interventions:**

Curative resection at the level of the linea alba on the bottom side was performed.

**Outcomes:**

No relapse has been observed since resection in either patient.

**Lessons:**

We herein report 2 cases of umbilical BCC with sweat glands and ducts. Although whether peri- and/or intra-tumor sweat gland structures are the source of the tumor or arise by transdifferentiation from tumor cells remains unclear, these findings may provide clues to help understand the morphopathogenesis of umbilical BCC in the future.

## 1. Introduction

Basal cell carcinoma (BCC) is the most frequent type of skin cancer, and occurs mostly in sun-exposed areas, rarely in the umbilicus.^[1]^ In particular, as far as we know, there have been few reports of umbilical BCC that mentioned sweat gland structure.^[2]^ BCC is considered to originate from either stem cells of the outer sheath of the hair follicle or the hair buldge.^[3]^ In addition, there is a hypothesis that all epidermal and adnexal epithelia are multipotent and that BCCs arise from these epithelia; however, this is not completely elucidated.^[4]^ Since BCC arising in the umbilicus without a hair follicle is relatively rare, we report 2 cases of umbilical BCC associated with intra- and peritumoral sweat gland structures.

## 2. Case presentation

### 2.1. Case 1

**Figure 1.**
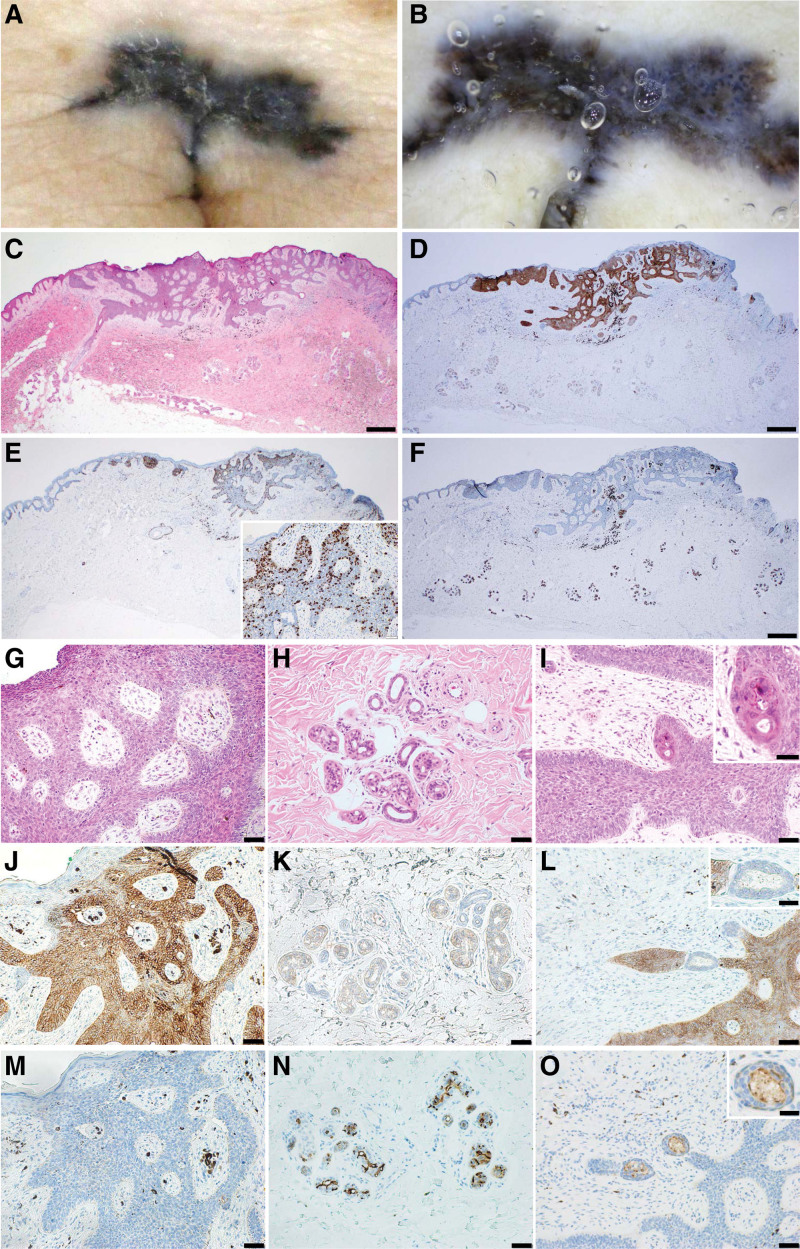
Case 1: (A) black plaque on the umbilicus. (B) Dermoscopy revealed multiple blue gray globules and large blue-gray ovoid nests. (C) Atypical cells contiguous with the epidermis forming a nest with a fenestrated arrangement at the margins (H&E, bar indicates 500 µm). (D–F) Immunohistochemical features of the tumor and sweat gland ducts around the tumor at low magnification. (D) The sweat gland ducts around the tumor were positive for Ber-EP4 (bar indicates 500 µm). (E) The MIB-1 index of the tumor was approximately 5%. (F) The sweat gland ducts were also positive for EMA (bar indicates 500 µm). (G–O) Histopathological findings and immunohistochemical staining (bar indicates 50 µm): tumor (G), sweat gland ducts around the tumor (H), gland ducts inside the tumor (I, inset; bar indicates 25 µm). (J–L) Ber-EP4: positive (J), weakly positive in sweat glands (K), negative (L, inset; bar indicates 25 µm). (M–O) EMA: negative (M), positive on the luminal side (N), positive on the luminal side (O, inset; bar indicates 25 µm). H&E = Hematoxylin and eosin staining.

The patient was a 61-year-old woman who visited our department 2 years previously who was aware of a black exudative plaque in her umbilicus. She had no history of surgery or trauma to the umbilicus. Clinical findings at the initial examination included a 20 × 3 mm large black plaque on the umbilicus (Fig. 1A). A dermoscopic examination revealed typical signs of BCC as multiple blue gray globules and large blue-gray ovoid nests (Fig. 1B). Plain computed tomography did not detect a ureteral remnant. A histopathological examination revealed atypical cells contiguous from the epidermis, which formed a nest with a fenestrated arrangement at the margins (Fig. 1C). An immunohistochemical analysis was performed using anti-epithelial antigen (Ber-EP4), anti-epithelial membrane antigen (EMA), and monoclonal antibody that recognized an epitope on Ki-67 (MIB-1) on BCC. An immunohistochemical analysis revealed Ber-EP4 (Fig. 1D) positivity and the MIB-1 index (Fig. 1E) was approximately 5%. In addition, EMA-positive glandular structures were seen within and around the tumor (Fig. 1F). At high magnification (Fig. 1G–O), the results of immunohistochemical staining of Ber-EP4 were as follows: the tumor cells were positive (Fig. 1J), the sweat gland cells around the tumor were weakly positive (Fig. 1K), and the ring-shaped glandular cells within the tumor were negative (Fig. 1L). The results of immunohistochemical staining of EMA were as follows: the tumor cells were negative (Fig. 1M), while the luminal side of sweat gland and duct cells (Fig. 1N), and glandular cells within the tumor (Fig. 1O) were positive.

According to these findings, we made a diagnosis of superficial basal cell carcinoma connected to sweat gland structures. No relapse was observed 10 months after curative resection at the level of linea alba on the bottom side.

### 2.2. Case 2

**Figure 2.**
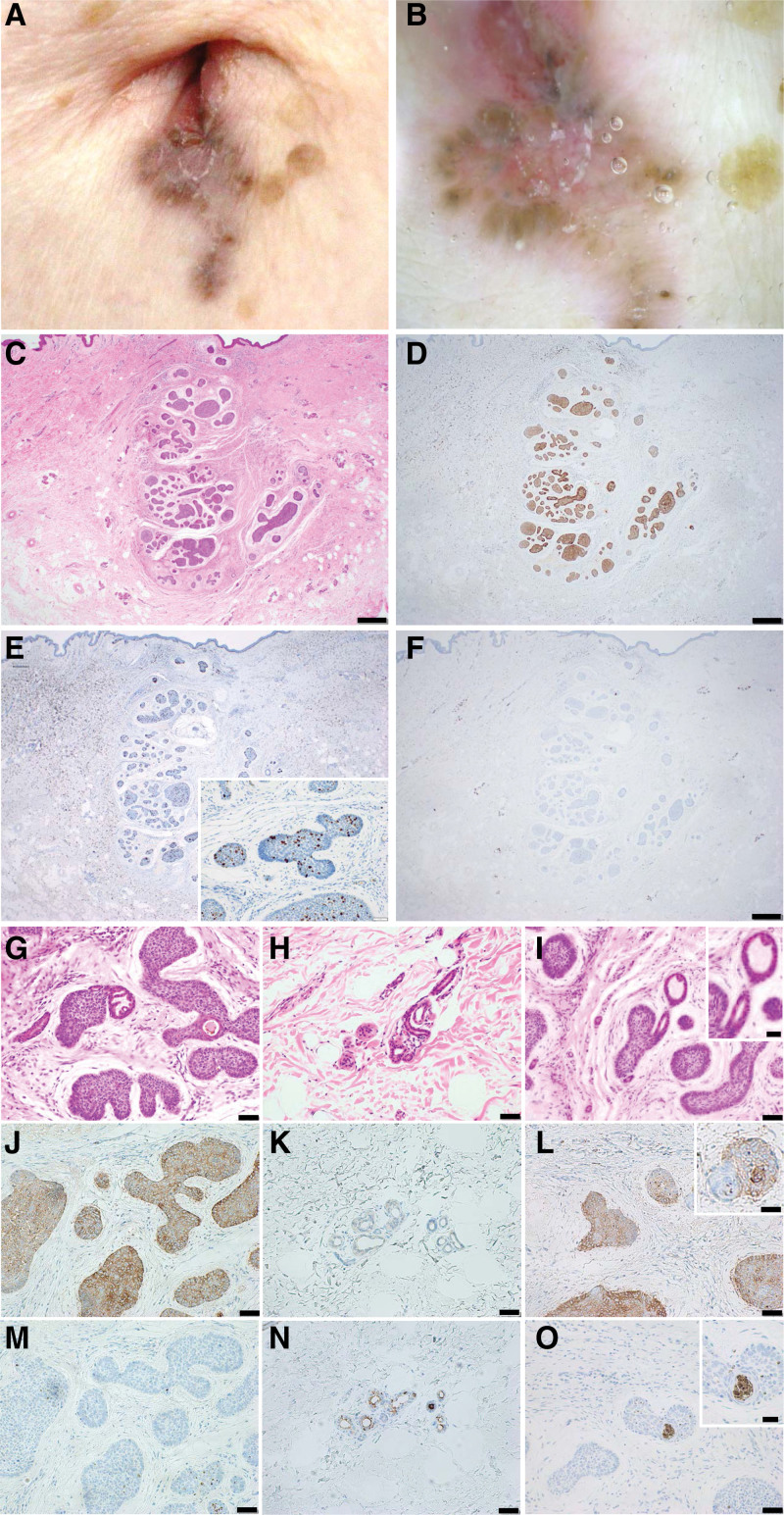
Case 2: (A) dark brown plaque on umbilicus. (B) Dermoscopy revealed large blue-gray ovoid nests and arborizing vessels. (C) Superficial basal cell carcinoma (H&E, bar indicates 500 µm). (D–F) Immunohistochemical features of the tumor and sweat gland ducts around the tumor at low magnification. (D) Sweat gland ducts around the tumor were positive for Ber-EP4 (bar indicates 500 µm). (E) The MIB-1 index of the tumor was approximately 5%. (F) Sweat gland ducts were also positive for EMA (bar indicates 500 µm). (G–O) Histopathological findings and immunohistochemical staining (bar indicates 50 µm): tumor (G), sweat gland ducts around the tumor (H), gland ducts inside the tumor (I, inset; bar indicates 25 µm). (J–L) Ber-EP4: positive (J), weakly positive in the sweat glands (K), negative (L, inset; bar indicates 25 µm). (M–O) EMA: negative (M), positive on the luminal side (N), positive on the luminal side (O, inset; bar indicates 25 µm). H&E = Hematoxylin and eosin staining.

An 80-year-old woman visited our department due to a dark brownish plaque in the umbilicus that had been present for 6 months prior to her first visit, with exudation 2 months prior to her first visit. She had no history of umbilical surgery or trauma. The clinical findings at the time of the initial examination were a dark brownish plaque 7 × 15 mm in diameter on the umbilicus (Fig. 2A). Large blue-gray ovoid nests and arborizing vessels were observed on dermoscopic examination (Fig. 2B). A diagnosis of superficial basal cell carcinoma was made based on the histopathological findings (Fig. 2C) and immunohistochemical staining of Ber-EP4 (Fig. 2D) and MIB-1 (Fig. 2E). In addition, EMA-positive gland duct structures presented around the tumor nest (Fig. 2F). At high magnification (Fig. 2G–O), the results of immunohistochemical staining of Ber-EP4 were as follows: the tumor cells were positive (Fig. 2J), the sweat gland cells around the tumor were weakly positive (Fig. 2K), and the glandular cells within the tumor were negative (Fig. 2L). The results of immunohistochemical staining of EMA were as follows: the tumor cells were negative (Fig. 2M), and the luminal side of sweat gland and duct cells (Fig. 2N), and the glandular cells within the tumor (Fig. 2O) were positive. No relapse was observed for 8 months after curative resection at the level of linea alba on the bottom side.

## 3. Discussion

Several previous studies investigated the origin site of BCC by morphological and immunohistochemical analyses.^[5–7]^ Although the previous studies hypothesized that BCC arises from embryonic primary epithelial germ cells, which have the potential to form hair and glandular epithelia, the evidence is not well established. In the present cases, umbilical BCC showed EMA-positive sweat gland structures in the peritumoral and intra-tumoral areas. There are several previous reports on sweat gland differentiation or development in BCC.^[2,8–12]^ Heenan and Bogle^[10]^ demonstrated the presence of eccrine differentiation in 16 of 66 BCCs and suggested that these BCCs might originate from the eccrine duct, including the acrosyringium, rather than the intervening epidermis. Only 1 study reported a case of BCC with gland and duct structures in the umbilicus.^[2]^ This case was classified as fibroepithelioma of Pinkus (FEP) variant BCC; thus, FEP may originate from the intraepidermal eccrine duct and have pluripotent capacity to differentiate toward sweat gland structures. Our cases could be distinguished FEP based on the clinical findings, and the fact that there was no evidence of the tumor spreading into the dermis via the eccrine ducts. In the present cases, sweat gland structures within the tumor demonstrated an EMA-positive staining pattern, as did the sweat gland duct structures surrounding the tumor. These results suggest that the glandular structures of BCC may originate from sweat glands and have the capacity to differentiate toward sweat gland structures in the umbilicus where no hair follicle exists. Whether peri-tumoral and/or intra-tumoral sweat gland structures are the source of tumors or whether they develop with trans-differentiation from tumor cells remains to be determined. A future omics analysis of tumor tissues and also neighbor resident cells may provide clues to understand the morpho-pathogenesis of umbilical BCC.

## Author contributions

**Conceptualization:** Yukiho Kurosaki, Saori Itoi-Ochi, Atsushi Tanemura.

**Investigation:** Yukiho Kurosaki, Asako Ota, Akiko Miyazaki, Kazunori Yokoi.

**Resources:** Hiroko Kato, Satoshi Nojima, Eiichi Morii.

**Supervision:** Eiichi Morii, Manabu Fujimoto, Atsushi Tanemura.

**Writing – original draft:** Yukiho Kurosaki, Saori Itoi-Ochi, Atsushi Tanemura.

**Writing – review & editing:** Yukiho Kurosaki, Saori Itoi-Ochi, Atsushi Tanemura.
